# A study on gait and muscle activation characteristics of persons with incomplete spinal cord injury with respect to age stratification

**DOI:** 10.3389/fbioe.2026.1743995

**Published:** 2026-02-04

**Authors:** Wen Liu, Fan Yang, Qing Yao, Han Xu, Dong Xu, Lifeng Qian

**Affiliations:** 1 Department of Rehabilitation, Zhejiang Chinese Medical University Affiliated Jiaxing Traditional Chinese Medicine Hospital, Jiaxing, Zhejiang, China; 2 Department of Encephalopathy, Zhejiang Chinese Medical University Affiliated Jiaxing Traditional Chinese Medicine Hospital, Jiaxing, Zhejiang, China; 3 Auckland Tongji Rehabilitation Medical Equipment Research Center, Tongji Zhejiang College, Jiaxing, Zhejiang, China

**Keywords:** age, AIS D level, gait analysis, incomplete spinal cord injury, surface electromyography

## Abstract

**Objective:**

To analyze the changes in gait and muscle activation characteristics between persons with incomplete spinal cord injury (SCI) and persons without SCI with respect to age stratification, and to examine the differences between these populations.

**Methods:**

Using the motion acquisition system and surface electromyography system, gait spatial-temporal, kinematic, dynamic parameters, and muscle activation characteristics were collected from 90 young, middle aged, and elderly persons with incomplete SCI, as well as an equivalent number of age-matched persons without SCI. The changes and differences in gait and muscle activation characteristics across age groups between these two populations were analyzed.

**Results:**

Compared to the controls, persons with incomplete SCI of different age showed reduced walking ability, with notable differences in stance phase, swing phase, double stance, step length, stride length and velocity (P < 0.05). The swing angle of knee flexion showed an increasing trend with age, while the swing angle of ankle inversion and abduction showed a decreasing trend with age. The average center of pressure (COP) velocity increased with age among SCI persons and COP path length of young SCI persons was shorter than that in middle aged and elderly persons. Furthermore, the pressure peak and the ratio of pressure onset time also increased with age. The muscle activation parameters indicated that a positive correlation with age was observed for the average power frequency of anterior tibialis, while no regular change with age was noted for the gastrocnemius medialis.

**Conclusion:**

Gait function and neuromuscular control strategies in persons with incomplete SCI are affected by age. The walking ability of young SCI persons was weaker compared to middle aged and elderly SCI persons with the same level, which may be related to differences in injury mechanism and age-specific expectations of walking ability. The changes in gait strategy and muscle activation patterns in persons with incomplete SCI provide an important basis for the development of age-specific rehabilitation intervention plans.

## 1 Introduction

Spinal cord injury (SCI) is a serious disabling condition (Liu J. et al., 2022; Li et al., 2023), often characterized by impairments of sensory, motor and autonomic nerve function. Compared to persons with complete SCI, those with incomplete SCI may gradually experience improvements in lower extremity walking function through targeted rehabilitation (Werner et al., 2021; Shank et al., 2019; Yuan et al., 2019). Reconstructing gait is a primary focus for persons with incomplete SCI undergoing rehabilitation. The process requires a detailed observation of gait patterns, a comprehensive understanding of gait characteristics, the identification of gait abnormalities, and the formulation of targeted corrections to develop scientifically-based walking rehabilitation strategies (Dambreville et al., 2019; Stampacchia et al., 2022; Herrera-Valenzuela et al., 2022; Zörner et al., 2022). Gait analysis is a scientific method that employs bio-mechanical techniques in order to quantitatively assess indicators, including kinematics, spatiotemporal and dynamics parameters during ambulation (Huang et al., 2022). Surface electromyography (sEMG) is considered a valuable indicator of muscle health and function, with its amplitude strongly correlated with strength and recovery. It is employed to assess the activities of multiple muscle groups engaged in complex movement tasks, such as daily activities, gait, or reaching and grasping actions. These methods are widely used in clinical practice and play an important role in gait reconstruction of SCI persons.

Current research on gait in SCI persons often focuses on the analysis of spatiotemporal parameters at varying injury levels and types (Baniasad et al., 2020; Eliseev et al., 2021; Marinho-Buzelli et al., 2019; Balbinot et al., 2021). Gait is influenced by various physiological factors, including age and gender. Researchers have recognized the necessity of considering the impact of age differences on gait in clinical studies. As age increases, walking ability becomes notably diminished compared to younger adults, characterized by decreased stride length, reduced speed, and shortened single stance time (Park et al., 2018; Motti Ader et al., 2021). Epidemiological evidence in 2022 has indicated that age is a risk factor for diseases related to SCI (Stewart et al., 2022). However, reports have not determined whether an intrinsic connection between walking ability and age among persons with incomplete SCI. Additionally, differences remain unclear in the spatiotemporal, kinematic, and dynamic gait characteristics among age groups of SCI persons.

This cross-sectional observational study characterizes gait and muscle activation patterns across age strata in persons with incomplete SCI. We compared spatiotemporal, kinematic, kinetic and electromyographic parameters between persons with incomplete SCI and neurologically intact controls, testing the hypothesis that young SCI persons demonstrated superior gait performance relative to their older counterparts. The overarching aim is to inform targeted rehabilitation strategies and provide a foundation for clinically applicable gait analysis.

## 2 Methods

### 2.1 Study design, setting, and participants

Between 2022 and 2024, 94 eligible persons with incomplete SCI were screened, and four declined participations, resulting in a final sample of 90 persons. All participants achieved functional ambulation following systematic inpatient rehabilitation. This standardized rehabilitation, typically spanning 3–6 months, integrated targeted exercise therapy, occupational therapy, and physical agent modalities to address specific functional deficits. According to internationally recognized age classification, those aged 20–39 were categorized as the young SCI group, those aged 40–59 as the middle-aged SCI group, and those aged 60–79 as the elderly SCI group.

The neurological examinations of all persons were performed according to the International Standards for Neurological Classification of Spinal Cord Injury (ISNCSCI), as established by the American Spinal Injury Association (ASIA). The level of SCI persons was assessed with respect to the neurological injury level (NIL) and key muscle strength, namely, the ASIA Impairment Scale (AIS) classification. The diagnostic criteria for persons with incomplete SCI included in this study were marked by AIS grade D, indicating preservation of motor function below the NIL, where more than half of the key muscles achieved grade 3 or above. The study excluded interference from lower limb spasms, thus ensuring compliance with the experimental requirements of the research.

The inclusion criteria for the SCI group were as follows: (a) participants aged 20–79 years; (b) stable vital signs; (c) persons with incomplete SCI at AIS grade D (Wedege et al., 2017); and (d) ability to walk independently or with the assistance of ankle-foot orthotics. Simultaneously, three groups of young, middle aged, and elderly persons, with the same number of cases and similar age to those in the SCI groups, were recruited from the outpatient department as control groups. The inclusion criteria were as follows: no history of SCI and normal walking function.

The exclusion criteria were as follows: (a) spinal instability or unhealed fractures of the pelvis and lower extremities; (b) severe low back pain or limited lumbar spine mobility; (c) complications such as orthostatic hypotension, heterotopic ossification, and deep vein thrombosis; (d) a modified Ashworth scale for lower extremity muscle tone of ≥1 (Liu H. et al., 2022); (e) the presence of electronic devices in the body, such as pacemakers, that may affect gait collection; and (f) cognitive impairment or refusal to cooperate.

Eligibility screening was conducted by a certified rehabilitation therapist based on completion of 3–4 months of conventional rehabilitation. Qualifying individuals received a full explanation of the study, and proceeded to gait and muscle activation testing in a timely manner. The study protocol followed the principles of the Declaration of Helsinki. It was approved by the ethics board of the China Rehabilitation Research Center (No. 2023-083-01) and registered in the Chinese Clinical Trials Registry (No. ChiCTR2400087701). Written informed consent was obtained from all the participants.

### 2.2 Instruments and variables

**FIGURE 1:**
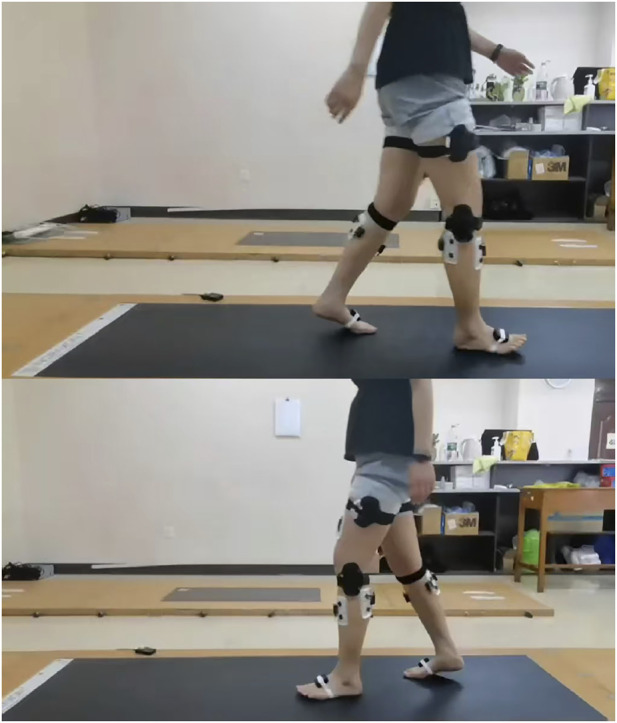
Location of gait sensor and sEMG sensor.

This study utilized the MyoMotion inertial sensor system (Noraxon, United States; sampling frequency, 100 Hz). Seven sensors were placed on specific anatomical points of the lower extremities, including the sacrum, both thighs, both calves, and both feet (Niswander et al., 2020; Rekant et al., 2022). Each sensor defined the middle joint and enabled the monitoring of joint angles and gait spatial parameters (Rantalainen et al., 2020) (Figure 1). The recorded position data were transmitted to the computer, and following zero-position calibration, the joint movements during walking were observed and documented (Bauer et al., 2024). Signal synchronization was facilitated by a wireless sEMG tester (Noraxon, USA; sampling frequency, 1,500 Hz). The skin of participants was cleansed and prepared accordingly. We analyzed the tibialis anterior (TA) and gastrocnemius medialis (GM), given their established critical role in gait mechanics following SCI.

The sEMG sensors were attached to the bulging areas of the TA and GM in accordance with guidelines from Surface Electromyography for the Non-Invasive Assessment of Muscles (Bao et al., 2024). Additionally, trunk muscles such as the rectus abdominis and erector spinae have been identified as potential biomarkers of inefficient central compensation after SCI (Shan et al., 2025). However, a conflict existed due to the special position of trunk muscles in relation to sensor attachment, therefore, these were not considered in this study.

Before the test commenced, the test process and precautions were explained to the participants to ensure they fully understanding and cooperation. All tests were administered by two rehabilitation physicians, with one responsible for collecting gait data and the other ensuring participants safety during walking. Participants were required to walk back and forth on the plantar pressure board at a natural speed four times to adapt to the walking environment before beginning the test. During the formal testing phase, participants were required to stand still for 10 s to calibrate posture and then walk back and forth on the plantar pressure board at a natural speed twice to acquire two sets of data. The walking test length was 6 m, and the plantar pressure board was centered (Niswander et al., 2020; Rantalainen et al., 2020). We focused on gait patterns and muscle activation under participants' natural speed. This speed reflects an energy-optimizing strategy that authentically reveals multi-joint coordination and intrinsic motor control. Assessing gait at this habitual speed minimizes kinematic artifacts from conscious pacing, thereby enhancing the reproducibility and clinical validity of our findings for quantifying functional impairment.

### 2.3 Data collection

Initially, the basic information of SCI persons and persons without SCI was recorded. Gait indicators were collected and analysed, including stance phase, swing phase, double stance phase, step length, and velocity, among others. The range of motion angles of the hip joint, knee joint and ankle joint were assessed. The center of pressure (COP) path length, average COP velocity, peak pressure were evaluated. Additionally, muscle activation parameters were examined, including the average power frequency (MPF) and median frequency (MF) of TA and GM during different walking cycles.

### 2.4 Statistical analysis

All the statistical analyses were performed using SPSS (version 25.0, IBM Corp., Armonk, NY, United States). Gait and sEMG data were analyzed using MATLAB (Waldon et al., 2023; Watanabe and Narouei, 2021; Acuña et al., 2019), and standardized parameters were obtained post noise reduction. The original sEMG data was processed by full wave rectification, smooth filtering and the application of root mean square (RMS) processing. The window constant was set to 200 ms (Liang et al., 2024). All RMS value in each gait cycle were calculated individually, and the average RMS was obtained following normalization. Consequently, the RMS distribution diagram within the gait cycle was generated, facilitating the calculation of the average standing RMS and swinging RMS.

The normally distributed data were represented by the means and standard deviations (SDs), while the intragroup and intergroup differences were assessed using one-way ANOVA. Skewed data were reported as the medians and interquartile distances, while the intragroup and intergroup differences were evaluated by rank-sum tests. Tukey’s test and Dunn-Bonferroni test were used for post hoc multiple comparisons (Szpala et al., 2022). P < 0.05 was considered statistically significant.

## 3 Results

According to the inclusion criteria, 30 cases of young, middle aged and elderly SCI persons were included in each category, and the control groups were constituted with young, middle aged and elderly persons without SCI. In accordance with the etiology classification outlined by the International Spinal Cord Injury Core Data set (version 3.0) (Biering-Sørensen et al., 2023), the causes of SCI among 90 persons were categorized as follows: 33 cases of fall (36.7%), 10 cases of transport (11.1%), 13 cases of tumor (benign/malignant) (14.4%), 9 cases of vascular etiology (10%), 6 cases of assault (6.7%), 4 cases of infection (4.4%) and 15 cases of other non-traumatic spinal cord dysfunction (16.7%), including 16 cases of high falling injury (17.8%) and 17 cases of fall down injury (18.9%). Among these, 26 persons had cervical SCI and 64 persons presented with thoracolumbar SCI (Table 1).

**TABLE 1:** Comparison of general data between young, middle aged and elderly SCI persons and control groups.

Baseline descriptive	Young SCI (a)	Middle aged SCI (b)	Elderly SCI (c)	Young control (d)	Middle aged control (e)	Elderly control (f)	X^2^/F	Adjusted P
Numbers	30	30	30	30	30	30	—	—
Gender (M: F)	20:10	21:9	18:12	21:9	8:22	8:22	—	—
Age (years)^#^	33.5 (8.0)^b,c,e,f^	50.5 (12.0)^c,d,f^	64.0 (7.0)^d,e^	33.5 (8.0)	51.5 (8.0)	65.5 (8.0)	158.903	0.000*
Etiology								
High falling injury	9	7	0					
Fall down injury	1	7	9					
Transport	7	3	0					
Tumor	7	4	2					
Assault	3	3	0					
Vascular etiology	2	2	5					
Infection	1	1	2					
Other non-traumatic spinal cord dysfunction	0	3	12	—	—	—	—	
Time since injury (months)^#^	6 (5)	6.5 (3)	6.5 (3)	—	—	—	—	—
NIL								
Cervical	9	9	8					
Thoracolumbar	21	21	22					
Lower extremity movement score^#^	40.0 (7)	38.5 (10)	40.0 (6)	—	—	—	4.206	0.122
Height (cm)^#^	173.0 (12.3)^e,f^	170.0 (9.0)	165.0 (14.3)^d^	173.0 (10.3)^e,f^	163.5 (9.8)	161.0 (7.5)	43.844	0.000*
Weight (kg)^#^	66.5 (25.1)	70.0 (15.0)	65.5 (14.3)	75.0 (25.5)	67.5 (17.8)	62.5 (18.3)	7.758	0.170
BMI^#^	23.0 (5.0)	24.0 (3.6)	24.0 (4.3)	23.8 (7.5)	25.6 (5.3)	24.2 (4.0)	7.853	0.165
The number of ankle-foot orthotics used	4	6	6	—	—	—	—	—

#:The skewed distribution data were reported as the median and interquartile distances.

SCI, spinal cord injury; M, F, Male, female; BMI, body mass index.

*indicated P < 0.05, which was considered to indicate statistical significance.

After multiple comparisons using Tukey’s test and Dunn-Bonferroni test, a-f indicated P < 0.05 when compared to group (a)-(f).

### 3.1 Gait spatiotemporal parameters

Compared to persons without SCI, SCI persons across different age strata exhibited varying degrees of reduced walking ability, with differences observed in six parameters, including stance phase, swing phase, double stance, step length, stride length and velocity (P < 0.05) (Table 2). Regarding velocity and cadence, the three groups of SCI persons increased with age. Compared to elderly SCI persons, a more pronounced weakening trend in velocity and cadence was exhibited by young SCI persons. Within the gait cycle, young SCI persons demonstrated larger bilateral stance phase and double stance compared to middle aged and elderly SCI persons, whereas their bilateral swing phase was observed to be smaller (Table 2).

**TABLE 2:** Comparison of gait spatiotemporal parameters between the young, middle aged and elderly SCI persons and control groups^#^.

Outcomes	Side	Young SCI (a)	Middle aged SCI (b)	Elderly SCI (c)	Young control (d)	Middle aged control (e)	Elderly control (f)	X^2^/F	Adjusted P
Stance phase (%)	Left	80 (13)^a,b,d,e,f^	79 (15)^d,e,f^	77 (14)^d,e,f^	69 (6)	68 (5)	67 (2)	72.249	0.000*
	Right	77. (13)^a,b,d,e,f^	77 (16)^d,e,f^	77 (11)^d,e,f^	68. (5)	67 (4)	66 (4)	90.535	0.000*
Swing phase (%)	Left	20 (13)^a,b,d,e,f^	21 (15)^d,e,f^	23 (14)^d,e,f^	31 (6)	32 (5)	33 (2)	72.249	0.000*
	Right	23 (13)^a,b,d,e,f^	23 (16)^d,e,f^	23 (11)^d,e,f^	32 (5)	33 (4)	34 (4)	90.535	0.000*
Double stance (%)		57 (23)^a,b,d,e,f^	57 (29)^d,e,f^	52 (28)^d,e,f^	36. (7)	34 (8)	33 (5)	95.480	0.000*
Foot rotation	Left	8.000 (8.450)	11.950 (8.200)^d^	9.550 (8.050)	7.450 (3.675)	8.000 (4.850)	8.300 (7.525)	11.840	0.037*
	Right	12.600 (10.450)	15.700 (8.000)	14.550 (7.500)	11.600 (7.125)	11.850 (5.875)	13.250 (8.625)	10.823	0.055
Step length (cm)	Left	27.500 (15.000)^a,b,d,e,f^	26.000 (14.000)^d,e,f^	27.000 (15.750)^d,e,f^	46.500 (8.000)	46.500 (12.500)	46.500 (11.500)	99.907	0.000*
	Right	27.000 (15.250)^a,b,d,e,f^	27.500 (11.250)^d,e,f^	28.500 (12.250)^d,e,f^	46.000 (10.750)	46.000 (15.250)	47.500 (7.000)	98.206	0.000*
Stride length (cm)		53.500 (27.500)^a,b,d,e,f^	55.500 (22.250)^d,e,f^	55.000 (27.250)^d,e,f^	95.000 (18.250)	90.000 (28.000)	94.500 (16.250)	103.650	0.000*
Step width (cm)		15.000 (6.500)^f^	16.000 (7.000)^f^	14.500 (5.250)^f^	14.000 (3.500)	13.000 (5.250)	11.000 (5.250)	20.712	0.033*
Velocity (km/h)		0.800 (1.130)^a,b,d,e,f^	0.900 (1.200)^d,e,f^	1.100 (0.750)^d,e,f^	2.350 (0.900)	2.300 (1.225)	2.550 (0.625)	97.964	0.000*
Cadence (steeps/min)		51.500 (38.50)^d,e,f^	67.500 (51.750)^e,f^	71.500 (31.500)^e,f^	82.500 (18.500)	89.000 (26.250)	92.500 (16.500)	61.719	0.000*
Time (m/s)	Left	1.145 (0.950)^a,b,d,e,f^	0.865 (0.760)^e,f^	0.910 (0.490)^e,f^	0.725 (0.163)	0.670 (0.185)	0.650 (0.120)	64.437	0.000*
	Right	1.215 (0.678)^a,b,d,e,f^	0.940 (1.000)^e,f^	0.820 (0.478)^e,f^	0.740 (0.203)	0.680 (0.228)	0.655 (0.120)	54.551	0.000*

#, The skewed distribution data were reported as the median and interquartile distances.

*indicated P < 0.05, which was considered to indicate statistical significance.

After multiple comparisons using Tukey’s test and Dunn-Bonferroni test, a-f indicated P < 0.05 when compared to group (a)-(f).

### 3.2 Gait kinematic parameters

**FIGURE 2:**
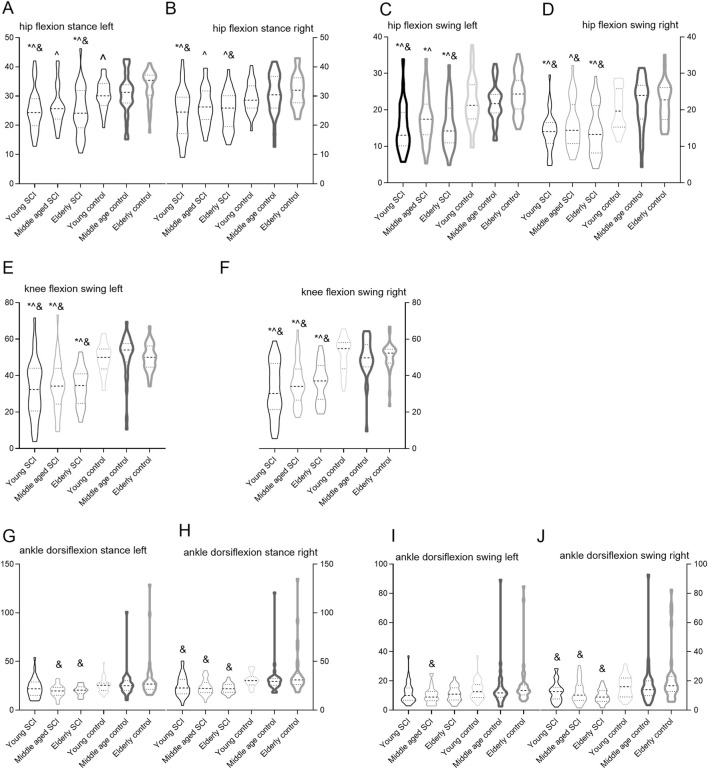
Comparison of hip flexion, knee flexion and ankle dorsiflexion angles among young, middle aged and elderly SCI persons and control groups. **(A)** hip flexion stance left; **(B)** hip flexion stance right; **(C)** hip flexion swing left; **(D)** hip flexion swing right; **(E)** hip flexion swing left; **(F)** hip flexion swing right; **(G)** ankle dorsiflexion stance left; **(H)** ankle dorsiflexion stance right; **(I)** ankle dorsiflexion swing left; **(J)** ankle dorsiflexion swing right. The violin plots described the median range of motion of the hip, knee, and ankle joints. The Y-axis represents the range of motion of the hip, knee, and ankle joints. * ^, & respectively indicate P < 0.05 when compared to young control, middle age control and elderly control.

Compared to the control groups, the hip, knee, and ankle joint range of motion in SCI persons showed an overall decreasing trend. During the stance phase, there were differences in the hip flexion and ankle dorsiflexion angles among SCI persons (P < 0.05). In young SCI persons, the hip flexion angle was smaller compared to middle aged and elderly SCI persons, while the ankle inversion and abduction angles were larger. During the swing phase, there were differences in the hip flexion, knee flexion, ankle dorsiflexion, ankle inversion, and ankle abduction angles (P < 0.05). The knee flexion angle among young SCI persons was smaller compared to middle aged and elderly SCI persons, while the ankle inversion and abduction angles were larger. No significant differences in other joint activities were noted (P > 0.05) (Supplementary Table S1). The swing angle of knee flexion showed an increasing trend with age of SCI persons, while the swing angles of ankle inversion and abduction decreased with age (Figure 2). In addition, the three groups of SCI persons showed an increase in the 95% confidence ellipse. The average COP velocity increased with age among SCI persons and the COP path length of young SCI persons was shorter than that in middle aged and elderly SCI persons (P < 0.05) (Supplementary Table S1).

### 3.3 Gait dynamic parameters

Compared to persons without SCI, the pressure peak among young, middle aged, and elderly SCI persons showed a decrease trend, alongside differences in the ratio of pressure onset time (P < 0.05) (Table 3). The pressure peak at the middle foot and the ratio of pressure onset time at the anterior foot increased with age among SCI persons. These observations indicated that the pressure distribution on the sole of young SCI persons was slightly worse compared to middle aged and elderly SCI persons, suggesting a weaker capacity to convert kinetic energy during walking. There were differences in the pressure peak at the anterior foot among the three groups of SCI persons (P < 0.05), but no consistent trend of increase or decrease was evident.

**TABLE 3:** Comparison of gait dynamic parameters between the young, middle aged and elderly SCI persons and control groups#.

Outcomes			Young SCI (a)	Middle aged SCI (b)	Elderly SCI (c)	Young control (d)	Middle aged control (e)	Elderly control (f)	X^2^/F	Adjusted P
Peak pressure (N)	The heel	Left	407.600 (180.350)	374.400 (161.975)	380.200 (151.625)	436.200 (134.075)	435.100 (169.675)	369.750 (133.150)	10.924	0.053
		Right	384.150 (211.400)	392.600 (137.675)	364.700 (131.900)	444.050 (169.600)	416.100 (139.600)	394.250 (100.025)	12.805	0.025*
	Middle foot	Left	77.300 (72.325)^a,b,d,e,f^	87.200 (98.575)^d,e,f^	111.300 (84.400)	177.000 (135.575)	156.700 (131.400)	163.900 (145.325)	30.945	0.023*
		Right	83.000 (84.075)^d^	97.600 (96.525)^d^	116.950 (84.850)	160.850 (165.975)	122.600 (85.950)	147.650 (119.100)	18.328	0.037*
	Anterior foot	Left	341.700 (300.850)^d,e,f^	318.450 (389.125)^d,e,f^	363.850 (192.475)^d,e,f^	647.800 (234.175)	617.400 (149.350)	577.550 (144.700)	69.630	0.000*
		Right	313.650 (326.050)^d,e,f^	263.550 (321.125)^d,e,f^	367.400 (215.500)^d,e,f^	701.850 (201.550)	631.400 (132.325)	580.400 (194.375)	89.781	0.000*
The ratio of pressure onset time	The heel	Left	1.483 ± 0.586	0.883 ± 0.337	0.867 ± 0.334	0.183 ± 0.051	0.117 ± 0.039	0.100 ± 0.037	8.935	0.112
		Right	1.633 ± 0.598^d^	0.483 ± 0.191	0.667 ± 0.388	0.483 ± 0.210	0.083 ± 0.035	0.117 ± 0.039	8.444	0.133
	Middle foot	Left	4.250 (5.500)	5.000 (6.500)	4.000 (5.625)	5.500 (5.250)	6.250 (3.125)	5.250 (3.125)	8.550	0.128
		Right	2.750 (5.375)	4.500 (5.500)	2.500 (4.125)^d,e^	5.750 (6.000)	7.000 (5.250)	5.750 (3.125)	20.729	0.022*
	Anterior foot	Left	0.500 (3.125)^a,b,d,e,f^	1.500 (3.125)^d,e,f^	1.500 (3.500)^d,e,f^	5.500 (5.625)	6.250 (2.250)	5.000 (2.625)	56.450	0.004*
		Right	1.250 (4.625)^d,e,f^	2.750 (4.625)^d,e,f^	1.750 (4.625)^e,f^	5.500 (6.125)	6.500 (3.125)	6.000 (2.500)	39.950	0.031*

#, The skewed distribution data were reported as the median and interquartile distances.

*indicates P < 0.05, which was considered to indicate statistical significance.

After multiple comparisons using Tukey’s test and Dunn-Bonferroni test, a-f indicated P < 0.05 when compared to group (a) - (f).

### 3.4 Muscle activation parameters

The muscle activation parameters of SCI persons were weaker compared to those of age-matched persons without SCI across time domain and frequency domain indicators. The activation parameters of TA indicated that the RMS of the right TA during the stance phase in young SCI persons was lower than in elderly SCI persons, with bilateral MPF showing a positive correlation with increasing age (P < 0.05). The activation parameters of the GM indicated that the RMS during the stance and swing phase in the three groups of SCI persons were lower than that in the control group (P < 0.05), but no consistent change with age was observed. No significant differences in bilateral MPF and MF of the GM were identified (P > 0.05) (Table 4).

**TABLE 4:** Comparison of muscle activation characteristics between the young, middle aged and elderly SCI persons and control groups.

Outcomes			Young SCI (a)	Middle aged SCI (b)	Elderly SCI (c)	Young control (d)	Middle aged control (e)	Elderly control (f)	X^2^/F	Adjusted P
TA	Left	Stance RMS (μV)	89.550 (121.650)	117.500 (95.275)	90.100 (95.375)	101.250 (66.425)	107.000 (82.550)	110.000 (79.225)	2.567	0.766
		Swing RMS (μV)	64.200 (82.225)	63.800 (60.400)	59.550 (54.950)	55.700 (41.325)	55.550 (41.725)	67.900 (27.325)	4.102	0.535
		MPF	86.450 (24.125)^d,e^	89.950 (26.450)^d,e^	95.750 (23.200)^d^	109.500 (39.275)	106.000 (29.025)	99.200 (30.450)	2.516	0.031*
		MF	60.300 (18.525)	70.600 (24.925)	73.700 (27.175)	79.750 (31.200)	77.150 (28.175)	71.900 (26.750)	10.312	0.067
	Right	Stance RMS (μV)	56.850 (50.575)^c^	95.650 (77.600)	120.000 (88.375)	100.150 (94.075)	80.050 (95.075)	82.950 (56.100)	14.039	0.018*
		Swing RMS (μV)	44.500 (71.100)	62.650 (36.675)	49.400 (102.675)	62.700 (53.300)	55.350 (68.575)	59.600 (50.600)	4.676	0.457
		MPF	88.900 (34.050)^d,e^	87.700 (30.225)^d,e^	89.050 (17.750)^d^	103.500 (24.725)	104.500 (44.775)	99.950 (50.125)	2.733	0.021*
		MF	66.350 (23.825)	68.700 (18.900)	68.500 (19.530)	74.100 (20.075)	81.300 (37.975)	74.350 (41.100)	7.427	0.191
GM#	Left	Stance RMS (μV)	112.000 (274.725)	80.100 (109.800)^d,e,f^	108.500 (231.975)	167.000 (130.750)	207.000 (168.500)	190.500 (456.000)	20.554	0.026*
		Swing RMS (μV)	44.750 (75.550)	26.450 (31.425)f	43.200 (87.725)	40.050 (91.625)	42.400 (50.800)	93.650 (237.675)	15.959	0.001*
		MPF	83.450 (29.925)	90.600 (40.250)	91.700 (31.100)	91.450 (37.000)	95.800 (34.725)	78.800 (44.325)	3.807	0.578
		MF	59.600 (24.650)	67.750 (38.800)	71.350 (27.225)	69.950 (34.000)	73.650 (25.375)	63.600 (42.250)	4.678	0.456
	Right	Stance RMS (μV)	61.050 (144.275)^d,f^	59.000 (105.325)^d,e,f^	100.300 (106.675)^d^	203.000 (214.000)	166.000 (135.625)	177.000 (147.375)	33.821	0.037*
		Swing RMS (μV)	34.100 (65.150)	20.800 (35.028)^d^	41.800 (36.300)	43.100 (111.150)	44.250 (45.900)	45.900 (173.900)	11.049	0.050*
		MPF	96.450 (28.650)	90.900 (24.700)	97.150 (24.475)	96.500 (34.175)	98.750 (22.475)	96.250 (45.825)	3.094	0.685
		MF	71.200 (28.975)	68.750 (31.250)	77.200 (26.200)	73.500 (31.375)	76.900 (21.250)	72.200 (36.825)	3.878	0.567

#, The skewed distribution data were reported as the median and interquartile distances.

RMS, root mean square; TA, tibial anterior; GM, gastrocnemius medialis; MPF, average power frequency; MF, median frequency.

*indicates P < 0.05, which was considered to indicate statistical significance.

After multiple comparisons using Tukey’s test and Dunn-Bonferroni test, a-f indicated P < 0.05 when compared to group (a) - (f).

## 4 Discussion

Current evidence lacks clear delineation of age-related differences in gait characteristics and muscle activation parameters among individuals with SCI. To address this, we recruited 90 persons with incomplete SCI and 90 age-matched non-SCI controls, stratified by age, and collected spatiotemporal, kinematic, kinetic, and electromyographic data during walking. Contrary to the expectation that young SCI persons would exhibit better walking function, our results demonstrated that young SCI persons had poorer gait performance compared to middle-aged and elderly counterparts. This finding contrasts with prior reports that unaided walkers show greater strength, speed, and lower metabolic cost than those using aids (Truong et al., 2025). In our cohort, the middle-aged and elderly SCI group had a higher usage rate of ankle-foot orthotics, but these groups exhibited better gait ability. Potential confounders such as gender, pain levels, body mass index (Truong et al., 2025), injury etiology, and walking expectations may underlie this discrepancy.

Current clinical guidelines emphasize that preventive and therapeutic strategies for SCI must account for population-specific injury characteristics, with rehabilitation plans tailored to the underlying trauma mechanism (La et al., 2023; Magg et al., 2024). In our cohort, injury etiology differed markedly by age: high-energy events such as falls from height and transportation accidents predominated in younger and middle-aged individuals, whereas low-energy falls were more common among the elderly (Yokota et al., 2024). Low-energy trauma typically induces less severe primary injury and fewer associated complications (Miyakoshi et al., 2021; Giraldo et al., 2021). In contrast, high-energy impacts often produce greater biomechanical disruption, concentrating stress on spinal ligaments and intervertebral discs and elevating risk for multisystem involvement. Consequently, even among individuals with comparable neurological levels, divergent injury mechanisms across age strata may explain observed variations in gait presentation.

Reduced gait velocity is a key predictor of lower extremity motor and balance function following neurological impairment. In individuals with SCI, disrupted neural drive compromises muscle activation amplitude and inter-muscular coordination. This deficit particularly weakens the hip extensors, hip flexors, and ankle plantar flexors, muscle groups critical for the stance-to-swing transition, thereby directly contributing to slowed walking (Lee et al., 2020). To maintain dynamic stability and ensure controlled forward progression of the center of mass, persons with SCI prolong their stance phase duration relative to able-bodied individuals. The marked differences in velocity and double support time observed between young persons with and without SCI reflect this significant functional decline (Gueugnon et al., 2019), which manifests clinically as muscle weakness, slowed movement initiation, and ambulation capacity loss (Kitamura et al., 2021). Although young persons with SCI often possess a higher premorbid functional baseline, recovery of walking after high-energy trauma is typically protracted. In contrast, elderly individuals with similar-severity, low-energy injuries may face a shorter gap between their post-injury capacity and lower pre-injury expectations. We therefore hypothesize that the non-linear relationship between age and walking ability after incomplete SCI may be partly explained by a discrepant alignment between actual function and walking expectations across age groups, with younger persons confronting a larger perceived functional gap.

Reduced extensibility of the muscle-tendon unit impairs movement control during gait, often manifesting as excessive hip and knee flexion in stance. To regain high-quality walking, younger individuals with SCI demonstrate a greater need for extensor muscle strength during stance compared to their elderly counterparts. This altered strategy increases energy absorption at the knee, while simultaneously reducing the contribution of ankle plantar flexors in swing (Gollie, 2018). To compensate for this distal weakness, proximal overuse of the hip extensors frequently occurs (Nadeau et al., 2011). Furthermore, an anteriorly displaced center of mass promotes a cautious shortening of stride length to mitigate fall risk (Haddas et al., 2018). Such gait deviations are reinforced by muscle-level biomechanical alterations, including excessive co-activation of antagonist muscles, which is a recognized contributor to gait and postural dysfunction in SCI. Collectively, these adaptations reflect a compensatory “stepping strategy” employed to offset diminished limb loading capacity and the loss of a swing phase (Wouda et al., 2024).

Beyond muscle weakness, restrictions in velocity and rhythm following incomplete SCI also stem from reduced joint range of motion. Limitations in hip, knee, and ankle mobility induce progressive physiological and mechanical alterations within the joints. Concurrent declines in motor and sensory function further reshape the kinematic trajectory of movement (Shin, 2022). Together, these factors represent genuine gait-limiting constraints and reflect age-dependent adaptations in neuromuscular control. Notably, during the swing phase, the overall knee flexion angle demonstrated a narrowing trend that correlated positively with age. Although reciprocal gait remained possible, altered flexion-extension amplitudes at the hip, knee, and ankle were apparent. This pattern suggests a compensatory strategy wherein increased flexion at the hip and knee is used to augment joint excursion, thereby promoting enhanced stability and balance during limb advancement.

While sex has been identified as a modest correlate of walking speed, with males typically walking faster (Truong et al., 2025), our study maintained strict inclusion/exclusion criteria that balanced key baseline characteristics like BMI. Nevertheless, an imbalanced gender ratio across age groups, a consequence of recruitment time constraints, may have influenced walking ability comparisons. Additionally, although participants completed four familiarization walks at a natural pace, the attachment of sensors and inertial measurement units to the lower limbs introduced a potential constraint. The inherent foreign body sensation and heightened caution likely prevented a fully natural walking pattern, a limitation acknowledged in prior research where fixed sensors can restrict movement and obscure accurate kinematic capture (Werner et al., 2023). This methodological factor is hypothesized to contribute to the observed gait differences between SCI and control groups. Such subjective influences may compromise analytical objectivity and increase data variability. Consequently, we suggest that clinical gait assessment integrate multimodal quantitative approaches. For instance, coupling biomechanical data with electroencephalography and functional near-infrared spectroscopy could elucidate cortical dynamics and neurovascular coupling during walking, thereby advancing future research into gait control and rehabilitation in SCI (Wu et al., 2025).

## 5 Conclusion

Gait function and neuromuscular control strategies in persons with incomplete SCI are affected by age. Divergent gait patterns were exhibited by persons across different age groups. The walking ability of young SCI persons was weaker compared to middle aged and elderly SCI persons with the same level. This discrepancy may be related to differences in injury mechanism and age-specific expectations of walking ability. The changes in gait strategy, balance control, and muscle activation patterns in persons with incomplete SCI provide an important bio-mechanical and neurophysiological basis for the development of age-specific rehabilitation intervention plans.

## 6 Limitations

This study has several limitations. Firstly, this study primarily focused on analyzing the gait and muscle activation characteristics of SCI persons across different age groups, without investigating other confounding factors affecting gait, such as spasms. Secondly, the gait behavior exhibited by SCI persons is highly individualized. Due to the inability of persons to perform multiple trials, only two trials were collected for each person. Further research involving a larger population is required to confirm the proposed results. In addition, there was inconsistency in the number of persons using ankle-foot orthotics collected in this study, which may have impacted the results. In the future, it is necessary to strictly control the number of persons using ankle-foot orthotics, or include those who can walk independently to improve result accuracy.

## Data Availability

The original contributions presented in the study are included in the article/Supplementary Material, further inquiries can be directed to the corresponding author.
