# Supplementary material for: A study on gait and muscle activation characteristics of persons with incomplete spinal cord injury with respect to age stratification
Source: Front Bioeng Biotechnol. 2026 Feb 4;14:1743995. doi: 10.3389/fbioe.2026.1743995 (PMC12913527; doi:10.3389/fbioe.2026.1743995)
Supplement: Supplementary file 1 [file Table1.docx]

Supplementary Material

# Supplementary Figures and Tables

## Supplementary Tables

**Supplementary Table 1.** Comparison of gait kinematic parameters between the young, middle aged and elderly SCI persons and control groups#

|  |  |  | young SCI  （a） | middle aged SCI（b） | elderly SCI  （c） | young control （d） | middle aged control  （e） | elderly control （f） | X^2^ | adjusted P |
| --- | --- | --- | --- | --- | --- | --- | --- | --- | --- | --- |
| hip flexion | stance | left | 24.350（9.250）^d,e,f^ | 25.700  （7.325）^f^ | 24.150（12.725）^d,e,f^ | 30.100  （7.550） | 31.300  （6.300） | 35.400（6.875） | 36.097 | 0.034* |
|  |  | right | 24.500（12.500）^d,e,f^ | 26.250  （9.800）^f^ | 25.900（10.580）^e,f^ | 28.600  （7.600） | 30.450  （10.850） | 32.000（8.550） | 27.917 | 0.043* |
|  | swing | left | 13.050（9.150）^d,e,f^ | 17.450  （8.400）^d,f^ | 14.200  （9.530）^d,e,f^ | 21.250  （9.425） | 21.700  （5.100） | 24.350（7.875） | 48.295 | 0.005* |
|  |  | right | 14.050（5.725）^d,e,f^ | 14.400（10.700）^e,f^ | 13.250（13.030）^d,e,f^ | 19.650  （10.525） | 23.950  （9.225） | 22.750（8.775） | 43.967 | 0.016* |
| hip abduction | stance | left | 9.125  （4.385） | 8.495  （5.913） | 9.705  （3.768） | 8.270  （5.323） | 9.925  （4.355） | 9.035  （4.605） | 3.004 | 0.699 |
|  |  | right | 8.095  （3.165） | 9.405  （5.108） | 8.545  （4.530） | 8.180  （5.423） | 9.355  （4.278） | 9.325  （4.250） | 2.860 | 0.722 |
|  | swing | left | 5.625  （3.705） | 5.050  （4.015） | 5.535  （3.715） | 6.090  （4.735） | 7.450  （5.475） | 6.230（3.9078） | 11.140 | 0.050 |
|  |  | right | 3.505  （3.885）^d,e^ | 5.870  （5.275） | 4.320  （3.328）^f^ | 6.945  （5.768） | 5.290  （6.010） | 7.170  （4.778） | 25.484 | 0.037* |
| hip rotation | stance | left | 12.800（8.973） | 13.100（10.593） | 12.200（10.195） | 9.795  （6.898） | 10.200  （3.423） | 10.850（5.530） | 7.474 | 0.188 |
|  |  | right | 12.250（9.020） | 10.650  （9.480） | 9.805  （7.173） | 10.350  （7.395） | 9.550  （4.450） | 10.055（5.598） | 7.066 | 0.216 |
|  | swing | left | 8.595  （5.668） | 7.840  （3.498） | 9.910  （7.198） | 9.145  （5.683） | 9.600  （4.265） | 9.925  （5.758） | 4.430 | 0.489 |
|  |  | right | 9.680  （7.220） | 8.565  （7.850） | 7.590  （5.588） | 9.000  （6.343） | 8.500  （4.808） | 9.500  （6.828） | 4.632 | 0.462 |
| knee flexion | stance | left | 35.520（17.750） | 35.900（12.750） | 36.350（10.200） | 36.500  （11.925） | 40.000  （8.700） | 37.050（11.325） | 5.496 | 0.358 |
|  |  | right | 36.700（16.400） | 37.200（14.525） | 37.500（13.525） | 38.550  （13.575） | 39.300  （11.525） | 38.500（10.550） | 6.766 | 0.239 |
|  | swing | left | 32.350（23.500）^d,e,f^ | 34.200（19.650）^d,e,f^ | 34.550（16.300）^d,e,f^ | 49.950  （10.950） | 54.050  （10.150） | 50.050（11.575） | 66.701 | 0.000* |
|  |  | right | 30.200（25.200）^d,e,f^ | 34.100（17.000）^d,e,f^ | 37.100（18.550）^d,e,f^ | 54.800  （14.425） | 49.750  （12.175） | 52.350（7.650） | 62.404 | 0.000* |
| ankle dorsiflexion# | stance | left | 21.900（13.725） | 19.650  （8.100）^f^ | 20.550  （6.975）^f^ | 25.550  （7.625） | 25.050  （10.175） | 26.800（11.800） | 25.853 | 0.047* |
|  |  | right | 22.850（14.900）^f^ | 22.350（10.675）^f^ | 21.900  （8.125）^f^ | 30.350  （8.550） | 29.550  （6.875） | 31.000（11.200） | 40.075 | 0.038* |
|  | swing | left | 10.055（8.390） | 8.960  （6.225）^f^ | 10.950  （8.385） | 12.650  （8.863） | 11.800  （7.183） | 13.450（6.878） | 12.803 | 0.027* |
|  |  | right | 12.700（8.013）^f^ | 10.315（10.418）^f^ | 8.980  （7.383）^f^ | 16.050  （13.013） | 14.050  （10.018） | 16.700（10.400） | 22.645 | 0.014* |
| ankle inversion | stance | left | 20.900（14.450） | 20.300（11.000） | 18.050  （8.600） | 21.300  （11.575） | 24.500  （13.350） | 19.550（10.325） | 8.390 | 0.136 |
|  |  | right | 14.550（10.548） | 15.500（10.773） | 12.650（10.635） | 13.850  （5.750） | 13.000  （7.743） | 17.400（10.125） | 10.234 | 0.069 |
|  | swing | left | 12.950（11.363） | 11.000  （8.790） | 7.370  （8.963）^e,f^ | 11.550  （8.768） | 13.700  （13.710） | 13.500（11.003） | 15.400 | 0.020* |
|  |  | right | 7.495  （4.883）^f^ | 7.895  （6.978）^f^ | 7.465  （5.528）^f^ | 11.900  （7.753） | 10.695  （7.808） | 16.700（11.825） | 30.505 | 0.000* |
| ankle abduction | stance | left | 12.350（7.975） | 12.200  （8.108） | 11.200  （5.788）^d^ | 13.200  （11.958） | 15.900  （11.990） | 15.800（11.800） | 10.253 | 0.068 |
|  |  | right | 12.250（7.405） | 11.400  （8.435） | 9.025  （5.173）^d^ | 13.050  （8.403） | 11.800  （8.570） | 11.500（9.065） | 14.614 | 0.006* |
|  | swing | left | 8.330  （8.078）^f^ | 8.020  （6.260）^f^ | 6.775  （6.073）^f^ | 10.750  （9.963） | 8.930  （9.480） | 10.550（9.728） | 23.132 | 0.024* |
|  |  | right | 6.530  （4.385）^f^ | 5.535  （7.473）^f^ | 4.565  （3.503）^d,e,f^ | 9.270  （6.603） | 8.355  （5.148） | 10.300（5.238） | 34.707 | 0.035* |
| 95% confidence ellipse | | | 820619.000（284496.750） | 855917.000（349153.500） | 877984.000（253239.000） | 861850.000  （318676.500） | 786736.500（306756.500） | 788425.000（275146.250） | 4.329 | 0.503 |
| COP path length | | | 2666.500（1129.250）^d,e,f^ | 2955.500（904.750）^d,e,f^ | 2791.500（990.500）^d,e,f^ | 2091.000（282.750） | 2102.000（423.500） | 2084.500（246.500） | 91.557 | 0.000* |
| average COP velocity | | | 147.000（96.000）^d,e,f^ | 176.500（101.500）^d,e,f^ | 178.500（85.000）^d,e,f^ | 258.000（73.500） | 252.500  （72.250） | 246.500（90.500） | 59.793 | 0.003* |

Note: #：The skewed distribution data were reported as the median and interquartile distances.

COP: Center of pressure.

* indicates P<0.05, which was considered to indicate statistical significance.

After multiple comparisons using Tukey's test and Dunn-Bonferroni test, a-f indicated P<0.05 when compared to group (a) - (f).
